# Mining The Cancer Genome Atlas gene expression data for lineage markers in distinguishing bladder urothelial carcinoma and prostate adenocarcinoma

**DOI:** 10.1038/s41598-021-85993-x

**Published:** 2021-03-24

**Authors:** Ewe Seng Ch’ng

**Affiliations:** 1 Advanced Medical and Dental Institute, Universiti Sains Malaysia, Bertam, Pulau Pinang Malaysia; 2 School of Medical and Life Sciences, Sunway University, Bandar Sunway, Selangor Malaysia

**Keywords:** Diagnostic markers, Tumour biomarkers

## Abstract

Distinguishing bladder urothelial carcinomas from prostate adenocarcinomas for poorly differentiated carcinomas derived from the bladder neck entails the use of a panel of lineage markers to help make this distinction. Publicly available The Cancer Genome Atlas (TCGA) gene expression data provides an avenue to examine utilities of these markers. This study aimed to verify expressions of urothelial and prostate lineage markers in the respective carcinomas and to seek the relative importance of these markers in making this distinction. Gene expressions of these markers were downloaded from TCGA Pan-Cancer database for bladder and prostate carcinomas. Differential gene expressions of these markers were analyzed. Standard linear discriminant analyses were applied to establish the relative importance of these markers in lineage determination and to construct the model best in making the distinction. This study shows that all urothelial lineage genes except for the gene for uroplakin III were significantly expressed in bladder urothelial carcinomas (p < 0.001). In descending order of importance to distinguish from prostate adenocarcinomas, genes for uroplakin II, S100P, GATA3 and thrombomodulin had high discriminant loadings (> 0.3). All prostate lineage genes were significantly expressed in prostate adenocarcinomas(p < 0.001). In descending order of importance to distinguish from bladder urothelial carcinomas, genes for NKX3.1, prostate specific antigen (PSA), prostate-specific acid phosphatase, prostein, and prostate-specific membrane antigen had high discriminant loadings (> 0.3). Combination of gene expressions for uroplakin II, S100P, NKX3.1 and PSA approached 100% accuracy in tumor classification both in the training and validation sets. Mining gene expression data, a combination of four lineage markers helps distinguish between bladder urothelial carcinomas and prostate adenocarcinomas.

## Introduction

Histological examination of a carcinoma from transurethral resection specimens, especially from the bladder neck, always triggers diagnostic consideration for the origin of the carcinoma as either bladder or prostate. The distinction is crucial as it impacts further management and prognosis. For advanced bladder urothelial carcinomas, the treatment options include neoadjuvant chemotherapy followed by cystectomy^1^, whereas for advanced prostate adenocarcinomas, the treatment options include radiotherapy and androgen deprivation therapy^2^.

For low-grade carcinomas, distinction between bladder urothelial carcinomas and prostate adenocarcinomas is usually possible based on morphological features. However, for high-grade bladder urothelial carcinomas and prostate adenocarcinomas, conclusive distinction based on morphology alone is difficult due to overlapping morphological features between these two types of carcinomas. In such cases, immunohistochemistry is performed, employing a panel of antibodies to interrogate the presence of certain proteins that act as urothelial lineage or prostate lineage markers^3^. A number of urothelial lineage markers such as GATA3 and p63, and prostate lineage markers such as prostate-specific antigen (PSA) and prostate acid phosphatase (PAP) are routinely used, acknowledging the variable sensitivities and specificities of these markers^4,5^.

For the past decades, the joint effort between the National Cancer Institute and the National Human Genome Research Institute has uncovered the genomic profiles of different types of cancers via large-scale genome sequencing and integrated multi-dimensional analyses. In particular, the Pan-Cancer analysis project under The Cancer Genome Atlas (TCGA) research network incorporates datasets across tumor types as well as across platforms by broad normalization efforts, enabling analyses for commonalities, differences and emergent themes^6^. Capitalizing on the publicly available transcriptomic data for bladder urothelial carcinomas and prostate adenocarcinomas, firstly, this study aims to verify that genes corresponding to urothelial lineage and prostate lineage markers employed in diagnostic immunohistochemistry are indeed significantly expressed in the corresponding groups of carcinomas. Secondly, this study aims to establish the relative importance of expressions of these genes in distinguishing between bladder urothelial carcinomas and prostate adenocarcinomas. Lastly, a model incorporating expressions of urothelial lineage and prostate lineage genes is constructed to best distinguish between bladder urothelial carcinomas and prostate adenocarcinomas.

## Methods

Using the Xena Browser online portal (https://xenabrowser.net/)^7^, TCGA Pan-Cancer database was filtered on primary tumor sites of bladder urothelial carcinoma or prostate adenocarcinoma. Lineage markers of contemporary diagnostic immunohistochemistry were pre-determined: GATA3, uroplakin III, thrombomodulin, p63, CK5/6, S100 calcium-binding protein P (S100P) and uroplakin II for urothelial lineage^5^, and prostate specific antigen (PSA), prostate-specific acid phosphatase (PSAP), prostein (P501S), prostate-specific membrane antigen (PSMA), NKX3.1, androgen receptor (AR), and alpha-methylacyl-CoA racemase (AMACR) for prostate lineage^4^. Gene expressions of these corresponding markers were downloaded, excluding cases without gene expression data. Relevant clinical data were downloaded from TCGA Prostate Cancer and TCGA Bladder Cancer databases.

Heat maps of these genes were drawn in Xena Browser. Differential gene expression analyses with RNA-seq data in unit log(TPM + 0.001) for these genes were performed between these two groups of carcinomas. Graphical display was done in R version 4.0.3 with the ggplot2 and ggpubr packages^8,9^. Welch-t test was applied in SPSS version 24.0. To address the multiple tests problematic, the significance level α was adjusted by the Bonferroni correction (α corrected = 0.05/14 tests = 0.003)^10^.

The cases were randomly divided into about 70% as the training set and the remaining as the validation set by randomly generated Bernoulli variates with probability parameter 0.7. To determine which gene expressions best distinguish between bladder urothelial carcinomas and prostate adenocarcinomas, standard linear discriminant analysis was performed in the training set and then validated in the validation set by SPSS version 24.0.

## Results

A total of 407 bladder urothelial carcinoma samples and 495 prostate adenocarcinoma samples were included in this study. Relevant clinical data of these bladder and prostate carcinoma samples are summarized in Table 1.

**Table 1:** Clinical characteristics of bladder urothelial carcinomas and prostate adenocarcinomas.

			Bladder urothelial carcinoma (n = 407)	Prostate adenocarcinoma (n = 495)
Age [years, mean (SD)]			68.1(10.6)	61.1(6.8)
Gender	Male		301 (74%)	495 (100%)
	Female		106 (26%)	–
Grade	Low		21 (5.2%)	–
	High		383 (94.1%)	–
	N/A		3 (0.7%)	–
Gleason Score	6		–	45 (9.1%)
	7		–	247 (49.9%)
	8		–	64 (12.9%)
	9		–	135 (27.3%)
	10		–	4 (0.8%)
T	0		1 (0.2%)	–
	1		3 (0.7%)	–
	2		119 (29.2%)	187 (37.7%)
	3		193 (47.5%)	291 (58.8%)
	4		58 (14.3%)	10 (2.0%)
	N/A		33 (8.1%)	7 (1.4%)
N	0		237 (58.2%)	343 (69.3%)
	1		46 (11.3%)	79 (16.0%)
	2		74 (18.2%)	–
	3		8 (2.0%)	–
	N/A		42 (10.3%)	73 (14.7%)
M	0		196 (48.2%)	453 (91.5%)
	1		11 (2.7%)	3 (0.6%)
	N/A		200 (49.1%)	39 (7.9%)

*N/A* not available.

**Figure 1:**
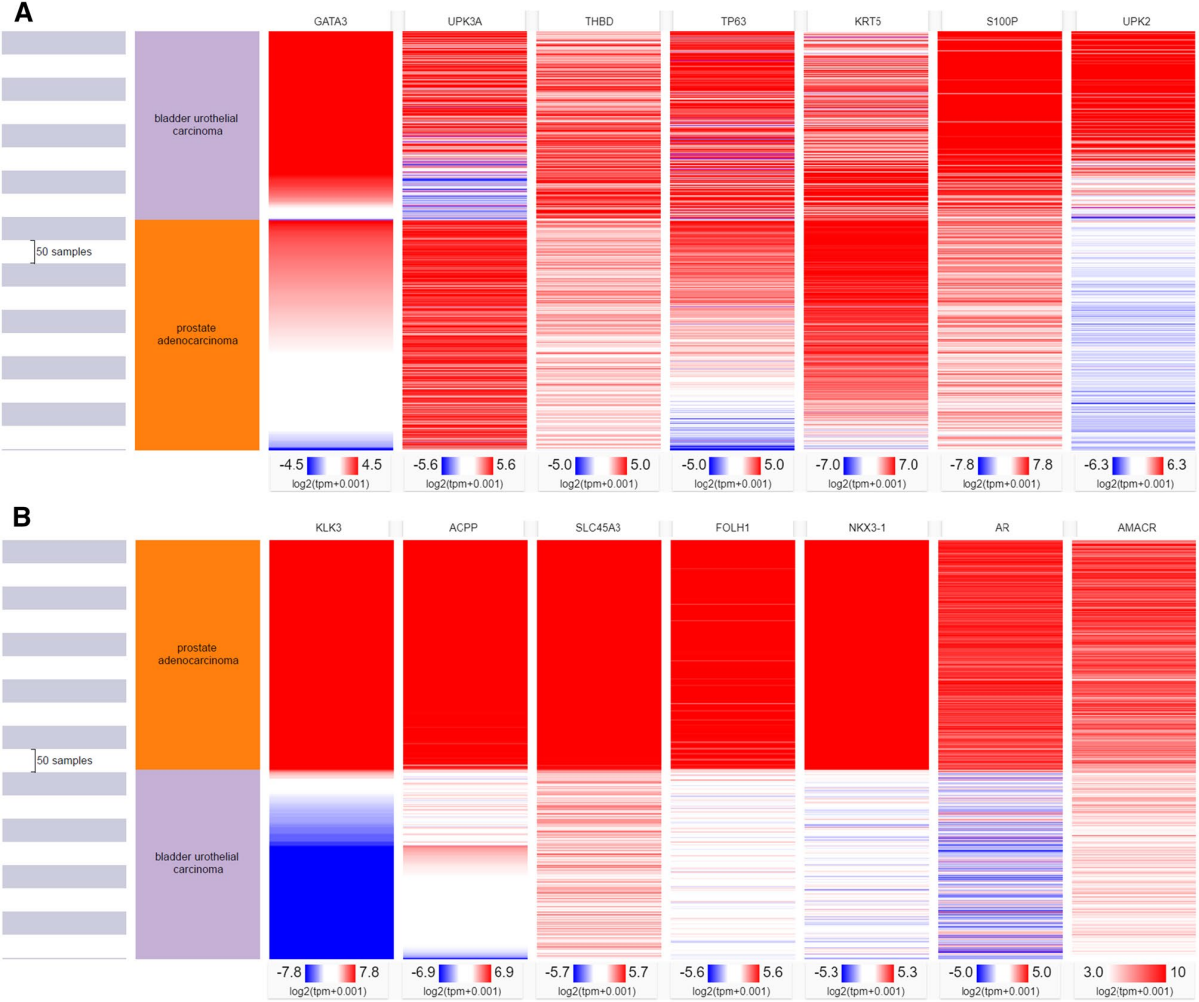
**(A)** Heat map for expressions of genes corresponding to the urothelial lineage markers (prepared using Xena Browser, accessed and analyzed online on 19 September 2020, https://xenabrowser.net/). **(B)** Heat map for expressions of genes corresponding to the prostate lineage markers (prepared using Xena Browser, accessed and analyzed online on 18 September 2020, https://xenabrowser.net/).

Heat map was drawn for expressions of genes corresponding to the urothelial lineage markers for both bladder urothelial carcinomas and prostate adenocarcinomas (Fig. 1A). The corresponding genes for GATA3, uroplakin III, thrombomodulin, p63, CK5/6, S100P and uroplakin II are *GATA3*, *UPK3A*, *THBD*, *TP63*, *KRT5*, *S100P* and *UPK2*, respectively. For CK5/6, only *KRT5* gene expression was included. Similarly, heat map for expressions of genes corresponding to the prostate lineage markers was drawn (Fig. 1B). The corresponding genes for PSA, PSAP, P501S, PSMA, NKX3.1, AR and AMACR are *KLK3*, *ACPP*, *SLC45A3*, *FOLH1*, *NKX3-1*, *AR* and *AMACR*, respectively.

**Figure 2:**
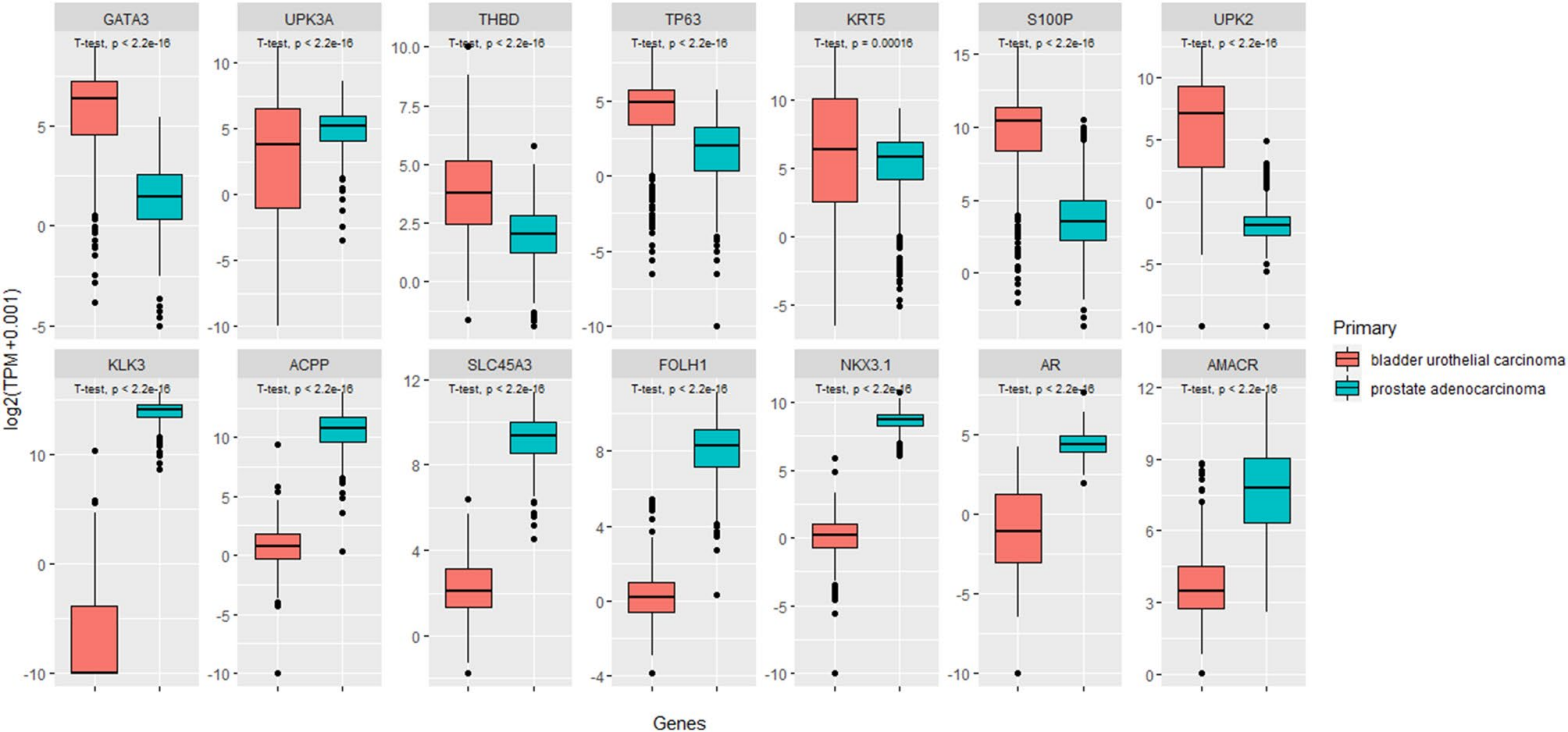
Differential gene expressions for urothelial and prostate lineage markers between bladder urothelial carcinomas and prostate adenocarcinomas (prepared using R version 4.0.3, https://cran.r-project.org/).

Figure 2 displays the boxplots of urothelial and prostate lineage gene expressions, comparing between bladder urothelial carcinomas and prostate adenocarcinomas. All urothelial lineage genes had significantly higher expressions in bladder urothelial carcinomas except *UPK3A*, which was significantly expressed in the prostate adenocarcinomas as compared to bladder urothelial carcinomas (all p < 0.001). All prostate lineage genes had significantly higher expressions in prostate adenocarcinomas as compared to those in bladder urothelial carcinomas (all p < 0.001).

Standard discriminant analysis was used to see if the model could predict the group membership of the dependent variable of either bladder urothelial carcinoma or prostate adenocarcinoma based on urothelial lineage gene expressions except *UPK3A*. This was first analyzed in the training set and then validated in the validation set. Table 2 shows the hit ratios for the training set and the validation set; predictive accuracies of the model for the training set and the validation set were 93.1% and 93.6% respectively. In descending order of importance for the urothelial lineage gene expressions, *UKP2*, *S100P*, *GATA3* and *THBD* were the most important predictors for bladder urothelial carcinoma based on the discriminant loading > 0.3 (Tables 3, 4).

**Table 2:** Hit ratios for the model based on urothelial lineage gene expressions.

	Actual	No of cases	Predicted group membership	
			Bladder urothelial carcinoma	Prostate adenocarcinoma
Training set	Bladder urothelial carcinoma	288	248	40
	Prostate adenocarcinoma	349	4	345
	Total	637		
Validation set	Bladder urothelial carcinoma	119	104	15
	Prostate adenocarcinoma	146	2	144
	Total	265		

93.1% of training set cases correctly classified.

93.6% of validation set cases correctly classified.

**Table 3:** Eigenvalues, canonical correlation and Wilk's lambda test of discriminant function based on urothelial lineage gene expressions.

Eigenvalues						Wilks' lambda			
Function	Eigenvalue	% of variance	Cumulative %	Canonical correlation, Rc	Rc^2^	Wilks' lambda	Chi-square	df	Sig
1	2.491	100	100	0.845	0.714	0.286	790.201	6	0.000

**Table 4:** Summary of interpretive measures for discriminant analysis based on urothelial lineage gene expressions.

Predictors	Bladder urothelial carcinoma (n = 288)		Prostate adenocarcinoma (n = 349)		F ratio	Discriminant loading (rank)	Unstandardized discriminant function coefficients	Standardized discriminant function coefficients
	Mean	SD	Mean	SD				
GATA3	5.668	2.315	1.381	1.706	721.891*	0.675(3)	0.142	0.284
THBD	3.578	1.883	1.984	1.202	167.453*	0.325(4)	0.266	0.412
TP63	3.849	2.871	1.579	2.518	112.922*	0.267(5)	− 0.112	− 0.300
KRT5	5.845	4.515	5.280	2.630	3.874^	0.049(6)	0.079	0.285
S100P	9.496	2.874	3.623	2.337	809.226*	0.715(2)	0.099	0.256
UPK2	6.016	4.276	− 1.897	1.504	1039.646*	0.811(1)	0.223	0.688
Constant							− 2.330	
Group centroids	1.735		− 1.432					

Discriminant function, D1 = 0.142(GATA3) + 0.266(THBD) − 0.122(TP63) + 0.079(KRT5) + 0.099(S100P) + 0.223(UPK2) − 2.33.

*p < 0.001.

^p = 0.049.

Similarly, standard discriminant analysis was performed based on prostate lineage gene expressions to see if the model could predict the group membership of the dependent variable of either bladder urothelial carcinoma or prostate adenocarcinoma. Table 5 shows the hit ratios for the training set and the validation set; predictive accuracies of the model for the training set and the validation set were 99.8% and 100.0% respectively. In descending order of importance for the prostate lineage genes, *NKX3-1*, *KLK3*, *ACPP*, *SLC45A3* and *FOLH1* were the most important predictors for prostate adenocarcinoma based on the discriminant loading > 0.3 (Tables 6, 7).

**Table 5:** Hit ratios for the model based on prostate lineage gene expressions.

	Actual	No of cases	Predicted group membership	
			Bladder urothelial carcinoma	Prostate adenocarcinoma
Training set	Bladder urothelial carcinoma	288	287	1
	Prostate adenocarcinoma	349	0	349
	Total	637		
Validation set	Bladder urothelial carcinoma	119	119	0
	Prostate adenocarcinoma	146	0	146
	Total	265		

99.8% of training set cases correctly classified.

100% of validation set cases correctly classified.

**Table 6:** Eigenvalues, canonical correlation and Wilk's lambda test of discriminant function based on prostate lineage gene expressions.

Eigenvalues						Wilks' lambda			
Function	Eigenvalue	% of Variance	Cumulative %	Canonical correlation, Rc	Rc^2^	Wilks' lambda	Chi-square	df	Sig
1	29.937	100	100	0.984	0.96826	0.032	2167.281	7	0.000

**Table 7:** Summary of interpretive measures for discriminant analysis based on prostate lineage gene expressions.

Predictors	Bladder urothelial carcinoma (n = 288)		Prostate adenocarcinoma (n = 349)		F ratio	Discriminant loading (rank)	Unstandardized discriminant function coefficients	Standardized discriminant function coefficients
	Mean	SD	Mean	SD				
KLK3	− 6.669	4.322	13.841	0.949	7427.176*	0.625(2)	0.148	0.444
ACPP	0.648	1.753	10.543	1.596	5546.788*	0.540(3)	0.151	0.252
SLC45A3	2.239	1.338	9.209	1.045	5444.004*	0.535(4)	0.233	0.276
FOLH1	0.253	1.333	7.989	1.498	4644.473*	0.494(5)	0.145	0.207
NKX3-1	− 0.061	1.614	8.654	0.685	8352.467*	0.663(1)	0.413	0.495
AR	− 0.908	2.703	4.398	0.782	1221.888*	0.254(6)	0.042	0.080
AMACR	3.702	1.429	7.640	1.836	883.260*	0.216(7)	− 0.034	− 0.057
Constant							− 5.490	
Group centroids	− 6.014		4.963					

Discriminant function, D1 = 0.148(KLK3) + 0.151(ACPP) + 0.233(SLC45A3) + 0.145(FOLH1) + 0.413(NKX3-1) + 0.042(AR) − 0.034(AMACR) − 5.49.

*p < 0.001.

Standard discriminant analysis was performed based on two most important urothelial lineage genes and two most important prostate lineage genes to see if the model could predict the group membership of the dependent variable of either bladder urothelial carcinoma or prostate adenocarcinoma. Table 8 shows the hit ratios for the training set and the validation set; predictive accuracies of the model for the training set and the validation set were 99.8% and 100.0% respectively. Prostate lineage genes of *NKX3-1* and *KLK3* appeared to be more important predictors as compared to urothelial lineage genes of *UPK2* and *S100P* (Tables 9, 10).

**Table 8:** Hit ratios for the model based on most important urothelial and prostate lineage gene expressions.

	Actual	No of cases	Predicted group membership	
			Bladder urothelial carcinoma	Prostate adenocarcinoma
Training set	Bladder urothelial carcinoma	288	287	1
	Prostate adenocarcinoma	349	0	349
	Total	637		
Validation set	Bladder urothelial carcinoma	119	119	0
	Prostate adenocarcinoma	146	0	146
	Total	265		

99.8% of training set cases correctly classified.

100% of validation set cases correctly classified.

**Table 9:** Eigenvalues, canonical correlation and Wilk's lambda test of discriminant function based on urothelial and prostate lineage gene expressions.

Eigenvalues						Wilks' lambda			
Function	Eigenvalue	% of variance	Cumulative %	Canonical correlation, Rc	Rc^2^	Wilks' lambda	Chi-square	df	Sig
1	25.095	100	100	0.981	0.96236	0.038	2064.693	4	0.000

**Table 10:** Summary of interpretive measures for discriminant analysis based on urothelial and prostate lineage gene expressions.

Predictors	Bladder urothelial carcinoma (n = 288)		Prostate adenocarcinoma (n = 349)		F ratio	Discriminant loading (rank)	Unstandardized discriminant function coefficients	Standardized discriminant function coefficients
	Mean	SD	Mean	SD				
UPK2	6.016	4.276	− 1.897	1.504	1039.646*	− 0.255(3)	− 0.096	− 0.297
S100P	9.496	2.874	3.623	2.337	809.226*	− 0.225(4)	− 0.017	− 0.043
NKX3-1	− 0.061	1.614	8.654	0.685	8352.467*	0.724(1)	0.512	0.614
KLK3	− 6.669	4.322	13.841	0.949	7427.176*	0.683(2)	0.230	0.689
Constant							− 3.202	
Group centroids	− 5.506		4.544					

Discriminant function, D1 = − 0.096(UPK2) − 0.017(S100P) + 0.512(NKX3-1) + 0.230(KLK3) − 3.202.

*p < 0.001.

## Discussion

To distinguish urothelial carcinomas from prostate adenocarcinomas, many studies have employed immunohistochemistry to investigate the use of several lineage markers. GATA3, Uroplakin III, Thrombomodulin, S100P, and Uroplakin II are commonly recommended as urothelial lineage markers^5^. Apart from that, urothelium expresses squamous cell-associated markers such as CK5/6 and p63; expressions of these markers are of value to distinguish from adenocarcinomas^5^. This study showed that genes corresponding to these urothelial lineage markers with the exception of *UPK3A* were indeed significantly expressed in the urothelial carcinomas as compared to those in prostate adenocarcinomas. Surprisingly, gene for uroplakin III, *UPK3A,* was highly expressed in prostate adenocarcinomas as compared to urothelial carcinomas. Contradictorily, by immunohistochemistry method, no expression of uroplakin III was observed in prostate adenocarcinomas across many studies^11–14^, yielding specificity of 100% in determining the origin as the bladder. This discrepancy between transcripts of *UPK3A* gene and uroplakin III protein expression in the prostate has been previously documented in a study^15^. Presence of *UPK3A* transcripts in the absence of uroplakin III protein is likely related to interactions between *UPK1B* gene expression and translation of *UPK3A* transcripts^15^.

Standard discriminant analysis of this study demonstrated that, in descending order of importance for the urothelial lineage markers, *UKP2, S100P, GATA3 and THBD* were the most important predictors for urothelial carcinoma by gene expression. These results corroborate to the studies whereby expressions of these urothelial lineage markers have been studied immunohistochemically^12,14,16,17^. Among these, GATA3 has been widely studied as a urothelial lineage marker and has a wide range of sensitivities (67–100%) across different studies^16^. Although most studies reported 0% staining in prostate adenocarcinomas, GATA3 generally lacks specificity because a variety of other tumors express this protein, especially breast carcinomas, cutaneous basal cell carcinomas, and trophoblastic and endodermal sinus tumors^18^. The corresponding protein for *UKP2*, uroplakin II, is a relatively new marker for urothelial lineage. The reported sensitivities and specificities for uroplakin II to differentiate urothelial carcinomas from prostate adenocarcinomas were 66–78% and 95–100%, respectively^12,19–21^. For S110P, the sensitivities and specificities were 71–100% and > 95% respectively in cases whereby antibody clone 16 was used ^16^. Thrombomodulin has been used as a urothelial lineage marker with sensitivities of 46–81% and specificity of 95–100% to differentiate from prostate adenocarcinomas^16,17^. Thrombomodulin also stains a small number of carcinomas from the lung, breast, ovary, and pancreas^14^.

On the other hand, recommended prostate lineage markers are PSA, PSAP, P501S, PSMA, NKX3.1, AR, and AMACR^4^. This study confirms that genes corresponding to these prostate lineage markers were indeed significantly expressed in the prostate adenocarcinomas as compared to those in urothelial carcinomas. Standard discriminant analysis of this study demonstrated that many of the prostate lineage markers genes were important predictors for prostate adenocarcinomas i.e. *NKX3-1, KLK3, ACPP, SLC45A3* and *FOLH1*, corresponding to NKX3.1, PSA, PSAP, P501S, and PSMA respectively. Among these, PSA is a sensitive and specific marker for the prostatic lineage with its sensitivities and specificities of 85–100% and 88–100%, respectively to differentiate from urothelial carcinomas^17^. PSAP is another conventional prostate lineage marker with high sensitivities and specificities of 92–95% and 81–100% respectively^17^. PSMA also has a similar range of sensitivities (87–100%) and specificities (83–100%) as a prostate lineage marker^3,17,22^. However, PSMA is also expressed in a few other tumor tissues such as squamous cell carcinomas and adenocarcinomas from stomach, colon and pancreas^22^. NKX3.1 and P501S are relatively newer prostate lineage markers. Sensitivities and specificities for NKX3.1 were 69–100% and 99–100%, and for P501S were 94–100% and 99–100%, respectively^3,17,23^. NKX3.1 is especially useful as it is expressed in many PSA-negative prostate adenocarcinomas^24^.

This study showed that by combination of four lineage markers with the highest discriminant loadings, i.e. *UKP2* and *S100P* for urothelial lineage and *NKX3-1* and *KLK3* for prostate lineage, classifications of training set and validation set approached 100% accuracies. Importantly, the prostate lineage genes took precedence over urothelial lineage genes as major predictors. Combination of NKX3.1, PSA, uroplakin II and S100P is therefore proposed to be the favored immunohistochemical test to resolve the dilemma of distinguishing between bladder urothelial carcinomas and prostate adenocarcinoma. This is in line with the recommendations provided by International Society of Urologic Pathology that combination of both lineage markers should be applied in such scenario with the weightage inclined towards prostate lineage markers^4^.

A few limitations of this study are acknowledged. Although findings of this study generally support the results of the previous studies, this study employed gene expression data of tumor tissue as compared to the visual evaluation of the lineage markers expressed on tumor cells by immunohistochemistry. Thus, discrepancy in expression between gene transcripts and proteins may arise as quantification of transcripts is dependent on tumor cellularity in the tumor tissue. Furthermore, in this study, 5.2% of bladder urothelial carcinomas were low grade and 9.1% of prostate adenocarcinomas had Gleason score of six. Inclusion of these low-grade carcinomas in this study as retrieved from the public databases differs from those studies focusing on high-grade carcinomas. Nevertheless, the findings of this study shall remain valid as total loss of expressions of all lineage markers in high-grade carcinomas is a rare event. Although this study readily provides combination of four lineage gene expressions as an algorithm to resolve the distinction between bladder urothelial carcinomas and prostate adenocarcinomas, transition to application by immunohistochemistry in routine diagnostic practice requires future validation.

## Conclusions

Data mining TCGA expression data for urothelial and prostate lineage markers, this study establishes that in descending order of importance, genes for uroplakin II, S100P, GATA3 and thrombomodulin are the most important urothelial lineage markers to distinguish a carcinoma as bladder urothelial carcinoma from prostate adenocarcinoma. In descending order of importance, genes for NKX3.1, PSA, PSAP, P501S and PSMA are the most important prostate lineage markers. Classification of a carcinoma of either bladder urothelial carcinoma or prostate adenocarcinoma reaches 100% accuracy by a combination of gene expressions of uroplakin II, S100P, NKX3.1, and PSA. This combination is readily applied in clinical diagnostic immunohistochemistry to resolve the dilemma in assigning the origin of a carcinoma as either bladder or prostate.

## Data Availability

The data of this study are available on public databases at Xena Browser online portal (https://xenabrowser.net/).

## References

[1] Witjes JA (2021). European Association of Urology guidelines on muscle-invasive and metastatic bladder cancer: Summary of the 2020 guidelines. Eur. Urol..

[2] Gillessen S (2020). Management of patients with advanced prostate cancer: Report of the advanced prostate cancer consensus conference 2019. Eur. Urol..

[3] Sanguedolce F (2019). Morphological and immunohistochemical biomarkers in distinguishing prostate carcinoma and urothelial carcinoma: A comprehensive review. Int. J. Surg. Pathol..

[4] Epstein JI, Egevad L, Humphrey PA, Montironi R (2014). Best practices recommendations in the application of immunohistochemistry in the prostate: Report from the International Society of Urologic Pathology consensus conference. Am. J. Surg. Pathol..

[5] Amin M, Trpkov K, Lopez-Beltran A, Grignon D (2014). Best practices recommendations in the application of immunohistochemistry in the bladder lesions: Report from the International Society of Urologic Pathology consensus conference. Am. J. Surg. Pathol..

[6] Weinstein JN (2013). The cancer genome atlas pan-cancer analysis project. Nat. Genet..

[7] Goldman MJ (2020). Visualizing and interpreting cancer genomics data via the Xena platform. Nat. Biotechnol..

[8] Wickham, H. *GGPLOT2: Elegant Graphics for Data Analysis 2016* (Springer, 2016).

[9] Kassambara, A. *ggpubr: ‘ggplot2’ Based Publication Ready Plots. R Package Version 0.4.0*. https://CRAN.R-project.org/package=ggpubr. (2020).

[10] Sedgwick P (2012). Multiple significance tests: The Bonferroni correction. BMJ.

[11] Kaufmann O, Volmerig J, Dietel M (2000). Uroplakin III is a highly specific and moderately sensitive immunohistochemical marker for primary and metastatic urothelial carcinomas. Am. J. Clin. Pathol..

[12] Smith SC (2014). Uroplakin II outperforms uroplakin III in diagnostically challenging settings. Histopathology.

[13] Moll R, Laufer J, Wu XR, Sun TT (1993). Uroplakin III, a specific membrane protein of urothelial umbrella cells, as a histological markers for metastatic transitional cell carcinomas. Verh. Dtsch. Ges. Pathol..

[14] Parker DC (2003). Potential utility of uroplakin III, thrombomodulin, high molecular weight cytokeratin, and cytokeratin 20 in noninvasive, invasive, and metastatic urothelial (transitional cell) carcinomas. Am. J. Surg. Pathol..

[15] Olsburgh J (2003). Uroplakin gene expression in normal human tissues and locally advanced bladder cancer. J. Pathol..

[16] Suryavanshi M (2017). S100P as a marker for urothelial histogenesis: A critical review and comparison with novel and traditional urothelial immunohistochemical markers. Adv. Anat. Pathol..

[17] Oh WJ (2016). Differential immunohistochemical profiles for distinguishing prostate carcinoma and urothelial carcinoma. J. Pathol. Transl. Med..

[18] Miettinen M (2014). GATA3: A multispecific but potentially useful marker in surgical pathology: A systematic analysis of 2500 epithelial and nonepithelial tumors. Am. J. Surg. Pathol..

[19] Hoang LL, Tacha D, Bremer RE, Haas TS, Cheng L (2015). Uroplakin II (UPII), GATA3, and p40 are highly sensitive markers for the differential diagnosis of invasive urothelial carcinoma. Appl. Immunohistochem. Mol. Morphol. AIMM.

[20] Tian W (2015). Utility of uroplakin II expression as a marker of urothelial carcinoma. Hum. Pathol..

[21] Li W (2014). Uroplakin II is a more sensitive immunohistochemical marker than uroplakin III in urothelial carcinoma and its variants. Am. J. Clin. Pathol..

[22] Mhawech-Fauceglia P (2007). Prostate-specific membrane antigen (PSMA) protein expression in normal and neoplastic tissues and its sensitivity and specificity in prostate adenocarcinoma: An immunohistochemical study using mutiple tumour tissue microarray technique. Histopathology.

[23] Chuang AY (2007). Immunohistochemical differentiation of high-grade prostate carcinoma from urothelial carcinoma. Am. J. Surg. Pathol..

[24] McDonald TM, Epstein JI (2021). Aberrant GATA3 staining in prostatic adenocarcinoma: A potential diagnostic pitfall. Am. J. Surg. Pathol..

